# Prognosis of late elderly patients with chronic hepatitis C after achieving a sustained viral response by direct‐acting antivirals

**DOI:** 10.1002/jgh3.12459

**Published:** 2020-11-23

**Authors:** Satoshi Takakusagi, Hitoshi Takagi, Takashi Kosone, Ken Sato, Satoru Kakizaki, Toshio Uraoka

**Affiliations:** ^1^ Department of Gastroenterology and Hepatology Kusunoki Hospital Fujioka Japan; ^2^ Department of Gastroenterology and Hepatology Gunma University Graduate School of Medicine Maebashi Japan; ^3^ Department of Clinical Research National Hospital Organization Takasaki General Medical Center Takasaki Japan

**Keywords:** direct‐acting antivirals, elderly, hepatitis C virus, hepatocellular carcinoma, prognosis

## Abstract

**Background and Aim:**

We investigated the prognosis of late elderly patients (≥75 years old) after the achievement of a sustained viral response (SVR) by direct‐acting antivirals (DAAs).

**Methods:**

One hundred and four late elderly patients and 251 young patients (≤74 years old) who had achieved an SVR were included. We compared the cumulative hepatocellular carcinoma (HCC) incidence rates and survival rates after DAA administration. Furthermore, the factors associated with HCC incidence and the causes of death after DAA administration were also investigated.

**Results:**

The cumulative HCC incidence rates for 1 and 3 years were 2.9% and 11.7% in the late elderly patients and 2.4% and 5.4% in the young patients, respectively. The cumulative survival rates for 1 and 3 years were 100% and 95.6% in the late elderly patients and 100% and 96.4% in the young patients, respectively, with no significant differences in those rates noted (*P* = 0.133, *P* = 0.322, respectively). In the late elderly patients, only a history of HCC was a significant factor associated with HCC incidence after DAA administration. Five late elderly patients died after achieving an SVR, and malignant liver tumor was the cause of death in three of those patients.

**Conclusions:**

The prognosis did not differ markedly between late elderly patients and young patients. The factor most strongly influencing the prognosis of late elderly patients was likely liver disease, including HCC. DAAs should be introduced even in late elderly patients who can be expected to have a relative long‐term survival.

## Graphical abstract

One hundred and four late elderly patients (75 years old) and 251 young patients (74 years old) who had achieved a sustained viral response by direct‐acting antivirals (DAAs) were included. The prognosis did not differ markedly between late elderly patients and young patients. The factor most strongly influencing the prognosis of late elderly patients was likely liver disease, including hepatocellular carcinoma. DAAs should be introduced even in late elderly patients who can be expected to have a relative long‐term survival. , Late elderly patients; , young patients.

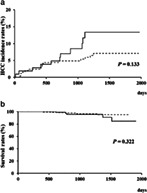

## Introduction

Direct‐acting antivirals (DAAs) have become the mainstream treatment for patients with chronic hepatitis C (CHC) worldwide. In Japan, glecaprevir/pibrentasvir (GLE/PIB) is now most prevalently used for the treatment of CHC, with high hepatitis C virus (HCV) eradication rates (>95%) among treatment‐naïve patients.^1^, ^2^ Furthermore, the introduction of sofosbuvir/velpatasvir (SOF/VEL) is expected to be highly effective in patients with decompensated cirrhosis.^3^ The combination therapy of SOF/VEL and ribavirin was reported to be effective even in patients who experienced virologic failure after DAAs.^4^

However, the aging of the population is progressing not only in Japan but around the world. In Japan, as of 1 February 2020, the number of people ≥75 years old (late elderly) is 18.55 million (14.7%) of the total population of 126 million, and the population 70–74 years old who will reach a late elderly state within 5 years is 8.92 million (7.1%).^5^ Asahina *et al*. reported that the cumulative and annual incidences of hepatocellular carcinoma (HCC) were significantly higher in older patients than in younger patients when stratified by the stage of fibrosis.^6^ Thus, elderly patients with CHC need more appropriate therapeutic intervention more urgently than younger patients.

Unlike interferon‐based treatments, DAAs are thought to be effective and tolerable even in elderly patients, similar to young patients.^7^ However, the benefits of DAAs for late elderly patients, like the suppression of HCC development or improvement of the prognosis, have not been analyzed in detail. We herein retrospectively compared late elderly CHC patients with young CHC patients (≤74 years old) for their prognosis after the achievement of a sustained viral response (SVR) by DAAs.

## Methods

### *Patients*

The 400 CHC patients who were treated with DAAs and achieved an SVR at 24 weeks after DAA administration (SVR24) until February 2020 in our hospital were potentially eligible for the study. Although all patients had been judged as cases without HCC development by imaging tests at the start of DAA administration, we excluded 24 who had started taking DAAs within 6 months after the last curative treatment of HCC (radiofrequency ablation [RFA], surgical resection, carbon ion radiotherapy, or liver transplantation). Furthermore, 21 patients in whom the fibrosis‐4 (FIB‐4) index of the end of DAAs could not be analyzed were also excluded. As a result, 104 late elderly patients and 251 young patients (total of 355 patients) were selected. The patients classified as Child‐Pugh class B or C were excluded from this study.

### *The surveillance and diagnosis of* *HCC*

Patients were basically assessed as outpatients every 4 weeks until 24 weeks after DAA administration. After that, the consultation interval for each patient was determined at the discretion of the attending physician according to the condition of each patient. After the start of DAA therapy, patients were examined with ultrasonography (US), contrast‐enhanced computed tomography (CT), or gadolinium‐ethoxybenzyl‐diethylenetriamine pentaacetic acid‐enhanced magnetic resonance imaging (EOB‐MRI) every three to 6 months until February 2020 for the surveillance of HCC. When questionable nodules were detected with US, an additional investigation with contrast CT or EOB‐MRI was performed for the definite diagnosis of HCC. The HCC diagnosis was confirmed with radiological findings diagnosed by board‐certified radiologists.

### *Demographic and laboratory data*

The demographic data, such as the sex, age (years), history of HCC, date of HCC development after the start of DAA, date of death, and laboratory data at the end of DAA administration, such as the platelet count, serum total bilirubin (T‐Bil), alanine aminotransferase (ALT), and aspartate aminotransferase (AST) levels, were included as baseline data. The FIB‐4 index was calculated as follows: (age × AST) / (platelet count × ALT^1/2^).

### *Virologic assessments*

HCV RNA was measured by serum HCV RNA on a polymerase chain reaction (PCR) assay using AccuGene m‐HCV (Abbott Japan, Tokyo, Japan; lower limit of quantification, 1.1 log IU/mL). An SVR24 was defined as continuously undetectable HCV RNA on a PCR assay until 24 weeks after the end of DAA administration.

### *Ethical considerations*

This study was approved by the institutional review board at our hospital, and the need for written informed consent was waived because of the retrospective nature of the study. In addition, this study complied with the Ethical Guidelines for Medical and Health Research Involving Human Subjects in Japan.

### *Statistical analyses*

Continuous variables were expressed as the median (interquartile range) and analyzed with the Mann–Whitney U test as nonparametric and unpaired analyses. Categorical variables were analyzed with Fisher's exact test. The cumulative incidence curve was determined with the Kaplan–Meier method, and differences among groups were assessed using the log‐rank test. A multivariate analysis was performed by a logistic regression analysis. All statistical analyses were performed using EZR version 1.41 (Saitama Medical Center, Jichi Medical University, Saitama, Japan).^8^

## Results

### *Baseline characteristics of the subjects*

The baseline characteristics of the 355 enrolled patients are shown in Table 1. The platelet count, serum albumin level, and the rates of male sex were significantly lower among the late elderly patients than the young patients (*P* = 0.002, *P* < 0.001, *P* = 0.045, respectively). Because age is one of the composing factors for the FIB‐4 index, the FIB‐4 index was significantly higher in the late elderly patients than in the young patients (*P* < 0.001). There were no significant differences in observation period, AFP levels, and history of HCC between the late elderly patients and the young patients.

**Table 1:** Baseline characteristics of the study patients (*n* = 355)

	Late elderly patients (*n* = 104)	Young patients (*n* = 251)	*P* value
Observation period (days)	818 (697–1302)	1036 (624–1340)	0.809
Age	79 (77–82)	65 (57–69)	<0.001
Male, *n* (%)	36 (34.6)	117 (46.6)	0.045
Platelet count (×10^3^/μL)	143 (109–205)	183 (133–225)	0.002
Total bilirubin (mg/dL)	0.8 (0.6–1.1)	0.8 (0.6–1.0)	0.658
ALT (IU/L)	18 (14–23)	18 (13–24)	0.786
Albumin (g/dL)	4.1 (3.8–4.3)	4.4 (4.0–4.6)	<0.001
AFP (ng/mL)	3.96 (0–6.68)	3.4 (2.41–5.31)	0.992
FIB‐4 index	3.3 (2.11–4.8)	1.86 (1.39–3.02)	<0.001
History of HCC, *n* (%)	6 (5.8)	12 (4.8)	0.791

AFP, α‐fetoprotein; ALT, alanine aminotransferase; FIB‐4, fibrosis‐4; HCC, hepatocellular carcinoma.

### *HCC* *incidence and survival in overall patients*

**Figure 1:**
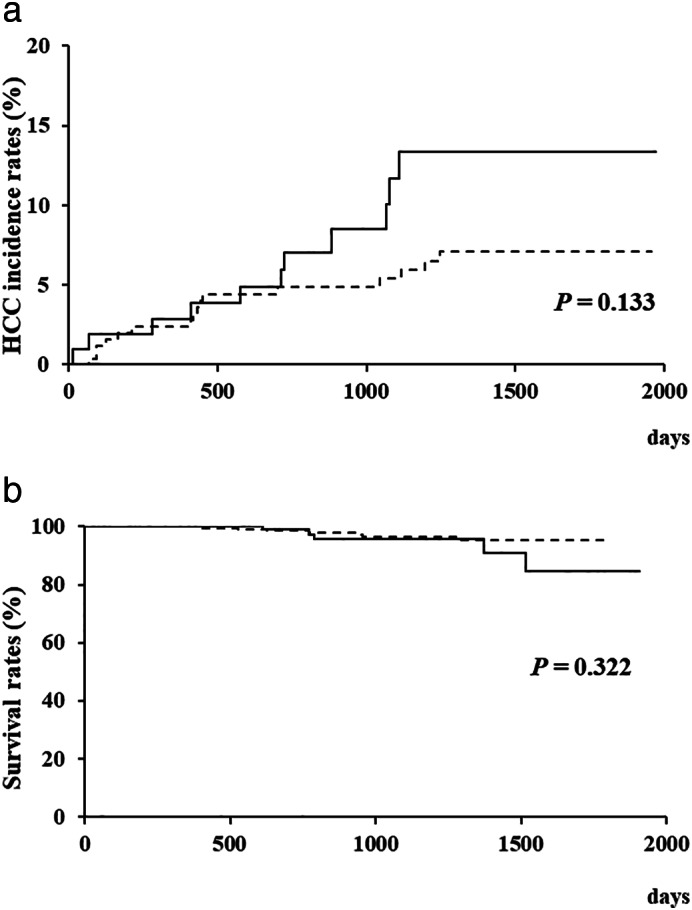
(a) Cumulative hepatocellular carcinoma (HCC) incidence rates of all patients. (b) Survival rates of all patients after the start of direct‐acting antivirals (DAAs). No significant differences between late elderly patients and young patients were demonstrated in either the cumulative HCC incidence rates or cumulative survival rates. 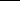, Late elderly patients; 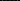, young patients.

During follow‐up, HCC developed in 11 (10.6%) of all late elderly patients and 16 (6.4%) of all young patients. The cumulative HCC incidence rates for 1 and 3 years were 2.9% and 11.7% in all late elderly patients and 2.4% and 5.4% in all young patients, respectively. No significant differences were demonstrated between those groups (*P* = 0.133) (Fig. 1a). The cumulative survival rates for 1 and 3 years were 100% and 95.6% in all late elderly patients and 100% and 96.4% in all young patients, respectively. Similarly to the HCC incidence, no significant differences in the cumulative survival rates were demonstrated between the groups (*P* = 0.322) (Fig. 1b). Figure S1 (Supporting information) showed the cumulative HCC incidence rates (A) and the cumulative survival rates (B) of the patients with HCC history. Figure S2 showed the cumulative HCC incidence rates (A) and the cumulative survival rates (B) of the patients without HCC history. There were no significant differences between the late elderly patients and the young patients. During the same period, five (4.8%) of the late elderly patients and seven (2.8%) of the young patients died. Of these, malignant liver tumor was the cause of death in three of the late elderly patients and two of the young patients (Table 2). Two of five late elderly patients and two of seven young patients had history of HCC before DAAs. Four late elderly patients and two young patients developed HCC after SVR. Five of seven HCC patients after SVR had history of HCC before DAAs.

**Table 2:** Characteristics of patients who died during observation period

	Age	Gender	Cause of death	Plt (×10^3^/μL)	T‐Bil (mg/dL)	ALT (IU/L)	FIB‐4 index	HCC history before DAAs	HCC incidence after SVR
1	63	M	Malignant lymphoma	73	0.8	26	4.74	Yes	Yes
2	66	M	ICC	110	0.8	20	4.03	No	No
3	68	F	Unknown	94	1.3	20	5.82	No	No
4	69	F	Sudden death	57	0.6	34	9.72	Yes	Yes
5	72	M	Pancreatic carcinoma	196	0.5	27	1.76	No	Yes
6	72	F	Sepsis^†^	168	0.7	13	3.23	No	No
7	73	M	HCC	214	0.5	13	2.47	No	Yes
8	77	F	ICC	161	1.1	18	2.92	No	No
9	77	M	MOF	157	1.3	18	4.74	No	Yes
10	79	F	Unknown	85	1.5	29	6.04	No	No
11	79	F	HCC	90	0.9	21	7.47	Yes	Yes
12	84	F	HCC	154	1.3	25	3.16	Yes	Yes

^†^ Sepsis due to urinary tract infection.

ALT, alanine aminotransferase; DAAs, direct‐acting antivirals; FIB‐4, fibrosis‐4; HCC, hepatocellular carcinoma; ICC, intrahepatic cholangiocarcinoma; MOF, multiple organ failure; Plt, platelet count; SVR, sustained viral response; T‐Bil, total bilirubin.

### *Factors associated with* *HCC* *incidence after* *DAA* *administration in overall patients*

During the follow‐up period, 27 (7.6%) of the 355 overall patients had HCC development after the start of DAA therapy. In a univariate analysis, the age, AFP, FIB‐4 index, and rates of an HCC history were significantly higher while the platelet count was significantly lower in the patients who had HCC development after DAA therapy than those who did not. In a multivariate analysis, the FIB‐4 index and history of HCC were the statistically significant predictive factors for HCC incidence after DAA administration (odds ratios: 1.31 and 28.2; 95% confidence intervals: 1.14–1.5 and 9.18–86.6, *P* < 0.001 and *P* < 0.001, respectively) (Table 3).

**Table 3:** Factors associated with HCC development after DAAs in overall patients (*n* = 355)

			Univariate analysis	Multivariate analysis	
	HCC (+) (*n* = 27)	HCC (−) (*n* = 328)	*P* value	Odds ratio (95% confidence interval)	*P* value
Age	73 (67–79)	68 (59–75)	0.006		
Male, *n* (%)	13 (48.1)	140 (42.7)	0.687		
Platelet count (×10^3^/μL)	119 (82–156)	174 (128–225)	<0.001		
Total bilirubin (mg/dL)	0.8 (0.6–1.3)	0.8 (0.6–1.0)	0.269		
ALT (IU/L)	21 (14–26.5)	18 (13–24)	0.204		
Albumin (g/dL)	4.1 (3.7–4.5)	4.3 (4.0–4.6)	0.036		
AFP (ng/mL)	5.36 (3.14–9.21)	3.35 (2.22–5.35)	0.048		
FIB‐4 index	4.4 (2.8–7.21)	2.08 (1.47–3.42)	<0.001	1.31 (1.14–1.5)	<0.001
History of HCC, *n* (%)	11 (40.7)	7 (2.1)	<0.001	28.2 (9.18–86.6)	<0.001

AFP, α‐fetoprotein; ALT, alanine aminotransferase; DAAs, direct‐acting antivirals; FIB‐4, fibrosis‐4; HCC, hepatocellular carcinoma.

### *Late elderly patients*

**Figure 2:**
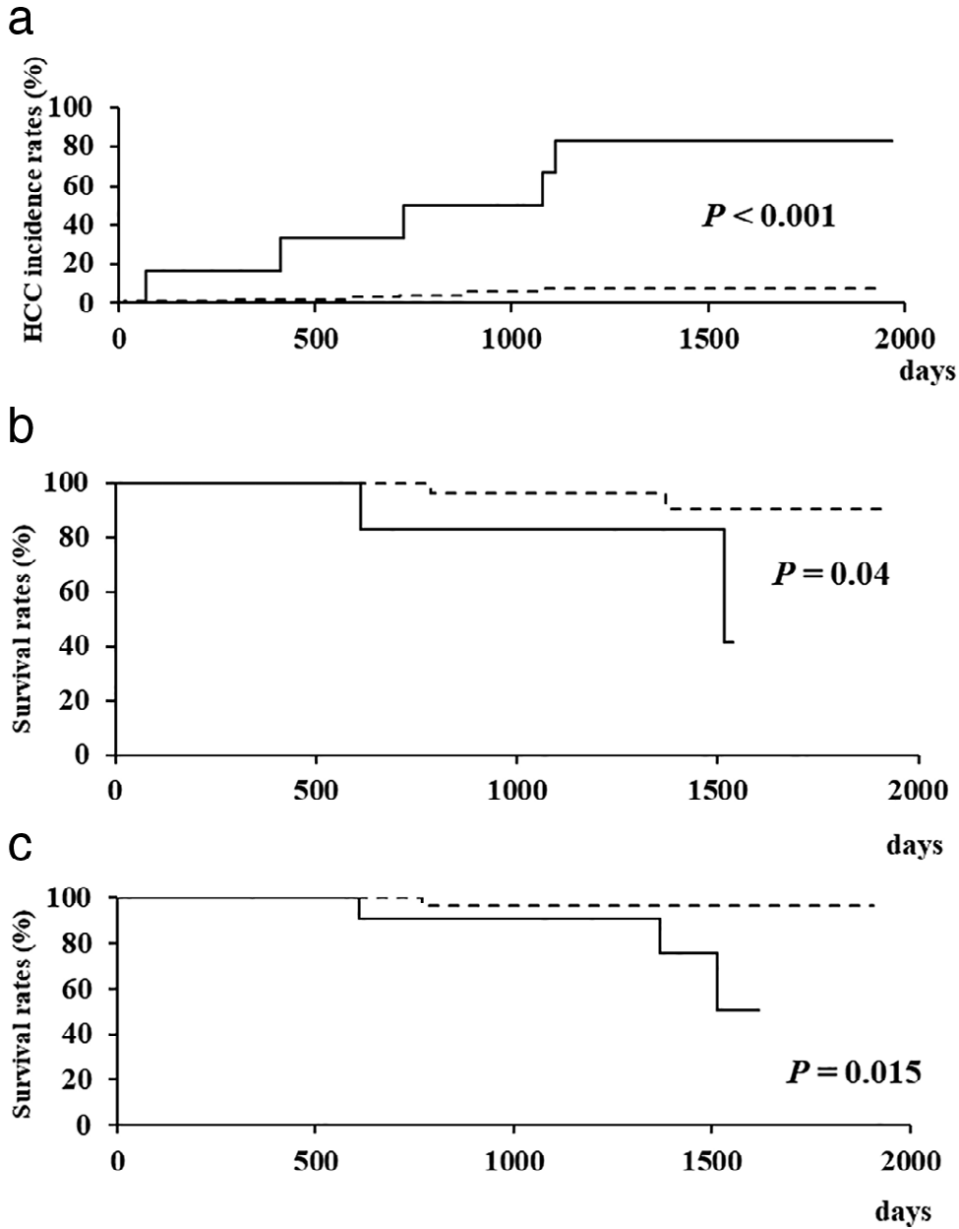
(a) Cumulative hepatocellular carcinoma (HCC) incidence rates. (b,c) The survival rates after the start of direct‐acting antivirals (DAAs) in late elderly patients. Significant differences in both the cumulative HCC incidence rates and survival rates were demonstrated between the patients with an HCC history and those without such a history (a,b). Furthermore, the cumulative survival rates of the patients who had HCC after DAA administration were significantly lower than those of the patients who did not (c). (a,b) 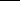, HCC history (+); 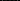, HCC history (−). (c) 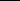, HCC incidence (+); 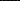, HCC incidence (−).

We also performed an analysis limited to the late elderly patients (*n* = 104). In the univariate analysis, only an HCC history was a statistically significant factor associated with HCC incidence after DAA administration (*P* < 0.001) (Table 4). Furthermore, we compared the characteristics between the patients with an HCC history (*n* = 6) and those without an HCC history (*n* = 98), but no significant differences were demonstrated (Table 5). During the follow‐up period, HCC recurred in five (83.3%) of the patients with an HCC history and newly developed in 6 (6.1%) of the patients without such a history. The cumulative HCC incidence rates for 1 and 3 years were 16.7% and 66.7% in the patients with an HCC history and 2.1% and 7.6% in those without such a history, respectively, showing a significant difference between those groups (*P* < 0.001) (Fig. 2a). The cumulative survival rates for 1 and 3 years were 100% and 83.3% in the patients with an HCC history and 100% and 96.4% in those without such a history. Similarly to the HCC incidence, a significant difference in the cumulative survival rates was demonstrated between those groups (*P* = 0.04) (Fig. 2b). Furthermore, we analyzed the association between the HCC incidence after DAA administration and the survival rates. The cumulative survival rates for 1 and 3 years were 100% and 90.9% in the patients who developed HCC after DAA therapy and 100% and 96.2% in those who did not, showing a statistically significant difference between the groups (*P* = 0.015) (Fig. 2c).

**Table 4:** Factors associated with HCC development after DAAs in late elderly patients (*n* = 104)

	HCC (+) (*n* = 11)	HCC (−) (*n* = 93)	*P* value
Age	82 (79–83)	79 (77–81)	0.125
Male, n (%)	4 (36.4)	32 (34.4)	1
Platelet count (×10^3^/μL)	131 (91–185)	144 (111–205)	0.41
Total bilirubin (mg/dL)	1.2 (0.7–1.3)	0.8 (0.6–1.0)	0.146
ALT (IU/L)	21 (12–26.5)	17 (14–22)	0.783
Albumin (g/dL)	4.0 (3.5–4.3)	4.1 (3.9–4.3)	0.455
AFP (ng/mL)	6.12 (2.8–9.75)	3.6 (0–6.3)	0.228
FIB‐4 index	4.47 (2.75–6.18)	3.26 (2.08–4.81)	0.119
History of HCC, n (%)	5 (45.5)	1 (1.1)	<0.001

AFP, α‐fetoprotein; ALT, alanine aminotransferase; DAAs, direct‐acting antivirals; FIB‐4, fibrosis‐4; HCC, hepatocellular carcinoma.

**Table 5:** A comparison of the late elderly patients with and without a history of HCC

	HCC history (+) (*n* = 6)	HCC history (−) (*n* = 98)	*P* value
Age	80 (79–87)	79 (77–81)	0.215
Male, *n* (%)	3 (50)	33 (33.7)	0.415
Platelet count (×10^3^/μL)	159 (117–201)	142 (109–204)	0.862
Total bilirubin (mg/dL)	0.9 (0.7–1.1)	0.8 (0.6–1.1)	0.872
ALT (IU/L)	20 (13–24)	18 (14–23)	0.955
Albumin (g/dL)	4.4 (4.3–4.7)	4.1 (3.8–4.3)	0.083
AFP (ng/mL)	7.94 (3.63–13.95)	3.36 (0–6.39)	0.111
FIB‐4 index	2.75 (2.34–6.09)	3.33 (2.09–4.81)	0.743

APP, α‐fetoprotein; ALT, alanine aminotransferase; FIB‐4, fibrosis‐4; HCC, hepatocellular carcinoma.

## Discussion

This study investigated the outcomes after achieving an SVR by DAAs in late elderly patients. The major finding from this study is that the prognosis after achieving an SVR by DAAs in late elderly patients was comparable to that in young patients.

Elderly patients are reportedly more likely to have advanced liver disease,^9^ including cirrhosis and HCC,^6^, ^10^, ^11^, ^12^ with an increased duration of infection than young patients. In our present study as well, the platelet count was lower in late elderly patient than in young patients. However, the HCC incidence rates of late elderly patients after DAAs were similar to those of young patients. Our analysis for the factors associated with HCC incidence after DAAs revealed that age was less significant than the FIB‐4 index or HCC history. Furthermore, although life expectancy generally becomes shorter as patients age, no marked difference in the survival rates after DAAs were observed between the late elderly patients and young patients in the present study.

SVR by DAA‐based regimen was reported to reduce the risk of developing HCC to 71% compared to treatment failure, which is a rate similar to that with interferon‐based treatment.^13^ Based on these findings, the suppressive effect of SVR by DAAs on the HCC incidence was considered obtainable even in late elderly patients, who tend to have more advanced fibrosis than young patients. We therefore believe that advanced age itself should not be considered a barrier to initiating DAAs, as previously reported.^14^

However, the maximum age at which patients should be treated by DAAs remains controversial. As the cost‐effectiveness of DAA therapy for elderly patients has been reported,^7^ we thought that patients with Child‐Pugh class A and those who were able to walk independently with a good general condition, like the subjects in our study, should be treated with DAAs in order to improve their prognosis and prevent them from infecting others.

It is also important to note that late elderly patients are often taking several medicines due to comorbidities. In such cases, it is necessary to consider the discontinuation or adjustment of some medicines in order to prevent drug–drug interactions before and during DAA therapy.^15^ Furthermore, it was also reported that the cumulative HCC incidence during the first 5 years after the completion of interferon therapy was similar between older SVR and non‐SVR patients.^6^ Based on these findings and the consideration of the cost‐effectiveness and adverse events, it was thought that DAAs should be introduced to patients who could be expected to have a reasonably good prognosis.

In our investigation limited to late elderly patients, the patients who developed HCC after DAA therapy had significantly lower cumulative survival rates than those who did not. Considering the causes of deaths, HCC incidence after achieving an SVR seemed to determine the prognosis even in late elderly patients. Tolerability of complications due to HCC treatment may be poorer in elderly patients than in young patients, so HCC treatment for elderly patients should be as minimally invasive as possible. The curability and safety of RFA treatment are definitive in cases with a small HCC in diameter,^16^ so early tumor detection is important. In the present study, only an HCC history before DAA therapy was a significant factor associated with HCC incidence after DAA administration in late elderly patients. Furthermore, the patients with an HCC history had a significantly higher cumulative HCC incidence and lower survival rates than those without an HCC history. Therefore, surveillance for HCC using detailed imaging tests, such as contrast‐enhanced CT or EOB‐MRI, in late elderly patients after achieving an SVR should be carefully continued, especially in cases with an HCC history.

Liver cirrhosis was reported to be significantly associated with hepatocarcinogenesis in elderly patients.^17^ In this study, the FIB‐4 index and history of HCC were significant predictors of the incidence of HCC after the administration of DAAs in the overall patient population. Although the FIB‐4 index tended to be higher in patients who developed HCC, a history of HCC only predicted the incidence of HCC in late elderly patients. This may be due to the small number of late elderly patients. At any rate, a history of HCC was strong predictor of the incidence of HCC in late elderly patients.

The present study has several limitations, such as its retrospective cohort nature, single‐institution setting, and relatively small number of late elderly subjects. As such, a further large‐scale and prospective study will be needed.

In conclusion, the prognosis after achieving an SVR by DAAs did not differ markedly between late elderly patients and non‐late elderly patients, and the factor most strongly influencing the prognosis in late elderly patients was liver disease itself, including HCC. Although patients with CHC should be treated early with DAAs, these agents can also be introduced to late elderly patients who missed receiving the treatment previously in order to improve their prognosis.

## Supporting information


**Figure S1** (A) Cumulative hepatocellular carcinoma (HCC) incidence rates of the patients with HCC history. (B) Survival rates of the patients with HCC history after the start of direct‐acting antivirals (DAAs). No significant differences between late elderly patients and young patients were demonstrated in either the cumulative HCC incidence rates or cumulative survival rates.

**Figure S2** (A) Cumulative hepatocellular carcinoma (HCC) incidence rates of the patients without HCC history. (B) Survival rates of the patients without HCC history after the start of direct‐acting antivirals (DAAs). No significant differences between late elderly patients and young patients were demonstrated in either the cumulative HCC incidence rates or cumulative survival rates.

## References

[1] Chayama K , Suzuki F , Karino Y *et al* Efficacy and safety of glecaprevir/pibrentasvir in Japanese patients with chronic genotype 1 hepatitis C virus infection with and without cirrhosis. J. Gastroenterol. 2018; 53: 557–65. 28948366 10.1007/s00535-017-1391-5 PMC5866824

[2] Toyoda H , Chayama K , Suzuki F *et al* Efficacy and safety of glecaprevir/pibrentasvir in Japanese patients with chronic genotype 2 hepatitis C virus infection. Hepatology. 2018; 67: 505–13. 28865152 10.1002/hep.29510 PMC5814891

[3] Curry MP , O'Leary JG , Bzowej N *et al* Sofosbuvir and velpatasvir for HCV in patients with decompensated cirrhosis. N. Engl. J. Med. 2015; 373: 2618–28. 26569658 10.1056/NEJMoa1512614

[4] Gane EJ , Shiffman ML , Etzkorn K *et al* Sofosbuvir‐velpatasvir with ribavirin for 24 weeks in hepatitis C virus patients previously treated with a direct‐acting antiviral regimen. Hepatology. 2017; 66: 1083–9. 28498551 10.1002/hep.29256

[5] Statistics Bureau of Japan . Population Estimates by Age (Five‐Year Groups) and Sex. Cited 1 Feb 2020. Available from http://www.stat.go.jp/english/index.htm

[6] Asahina Y , Tsuchiya K , Tamaki N *et al* Effect of aging on risk for hepatocellular carcinoma in chronic hepatitis C virus infection. Hepatology. 2010; 52: 518–27. 20683951 10.1002/hep.23691

[7] Rheem J , Sundaram V , Saab S . Antiviral therapy in elderly patients with hepatitis C virus infection. Gastroenterol. Hepatol. 2015; 11: 294–346. PMC4962680 27482173

[8] Kanda Y . Investigation of the freely available easy‐to‐use software 'EZR' for medical statistics. Bone Marrow Transplant. 2013; 48: 452–8. 23208313 10.1038/bmt.2012.244 PMC3590441

[9] Marcellin P , Asselah T , Boyer N . Fibrosis and disease progression in hepatitis C. Hepatology. 2002; 36: S47–56. 12407576 10.1053/jhep.2002.36993

[10] Tong MJ , el‐Farra NS , Reikes AR , Co RL . Clinical outcomes after transfusion‐associated hepatitis C. N. Engl. J. Med. 1995; 332: 1463–6. 7739682 10.1056/NEJM199506013322202

[11] Davila JA , Morgan RO , Shaib Y , McGlynn KA , El‐Serag HB . Hepatitis C infection and the increasing incidence of hepatocellular carcinoma: a population‐based study. Gastroenterology. 2004; 127: 1372–80. 15521006 10.1053/j.gastro.2004.07.020

[12] El‐Serag HB , Kanwal F , Richardson P , Kramer J . Risk of hepatocellular carcinoma after sustained virological response in Veterans with hepatitis C virus infection. Hepatology. 2016; 64: 130–7. 26946190 10.1002/hep.28535 PMC4917456

[13] Ioannou GN , Green PK , Berry K . HCV eradication induced by direct‐acting antiviral agents reduces the risk of hepatocellular carcinoma. J. Hepatol. 2018; 68: 25–32. 10.1016/j.jhep.2017.08.030 PMC5837901 28887168

[14] Su F , Beste LA , Green PK , Berry K , Ioannou GN . Direct‐acting antivirals are effective for chronic hepatitis C treatment in elderly patients: a real‐world study of 17 487 patients. Eur. J. Gastroenterol. Hepatol. 2017; 29: 686–93. 28195877 10.1097/MEG.0000000000000858 PMC6534142

[15] Ippolito AM , Iacobellis A , Milella M *et al* Hepatitis C virus clearance in older adults. J. Am. Geriatr. Soc. 2018; 66: 85–91. 29135030 10.1111/jgs.15140

[16] Livraghi T , Meloni F , Di Stasi M *et al* Sustained complete response and complications rates after radiofrequency ablation of very early hepatocellular carcinoma in cirrhosis: Is resection still the treatment of choice? Hepatology. 2008; 47: 82–9. 18008357 10.1002/hep.21933

[17] Kumada T , Toyoda H , Kiriyama S *et al* Characteristics of elderly hepatitis C virus‐associated hepatocellular carcinoma patients. J. Gastroenterol. Hepatol. 2013; 28: 357–64. 23190084 10.1111/jgh.12057

