# Supplementary figures and images for: Prognosis of late elderly patients with chronic hepatitis C after achieving a sustained viral response by direct‐acting antivirals
Source: JGH Open. 2020 Nov 23;5(1):122–7. doi: 10.1002/jgh3.12459 (PMC7812467; doi:10.1002/jgh3.12459)

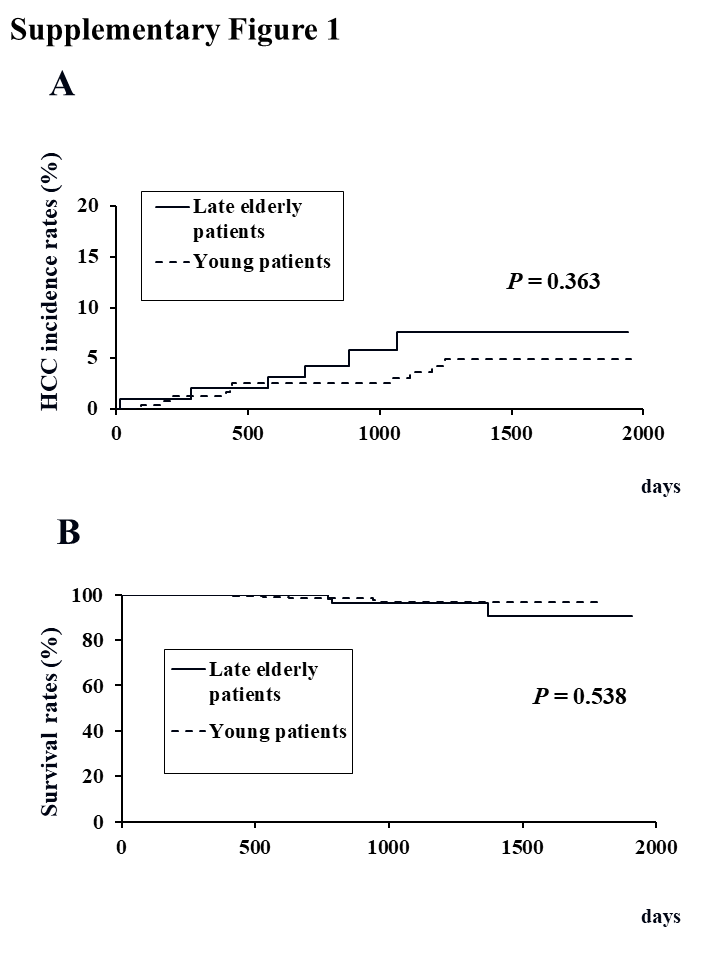

Supplement: Supplementary file 1 — Figure S1 (A) Cumulative hepatocellular carcinoma (HCC) incidence rates of the patients with HCC history. (B) Survival rates of the patients with HCC history after the start of direct‐acting antivirals (DAAs). No significant differences between late elderly patients and young patients were demonstrated in either the cumulative HCC incidence rates or cumulative survival rates. [file JGH3-5-122-s001.TIF]

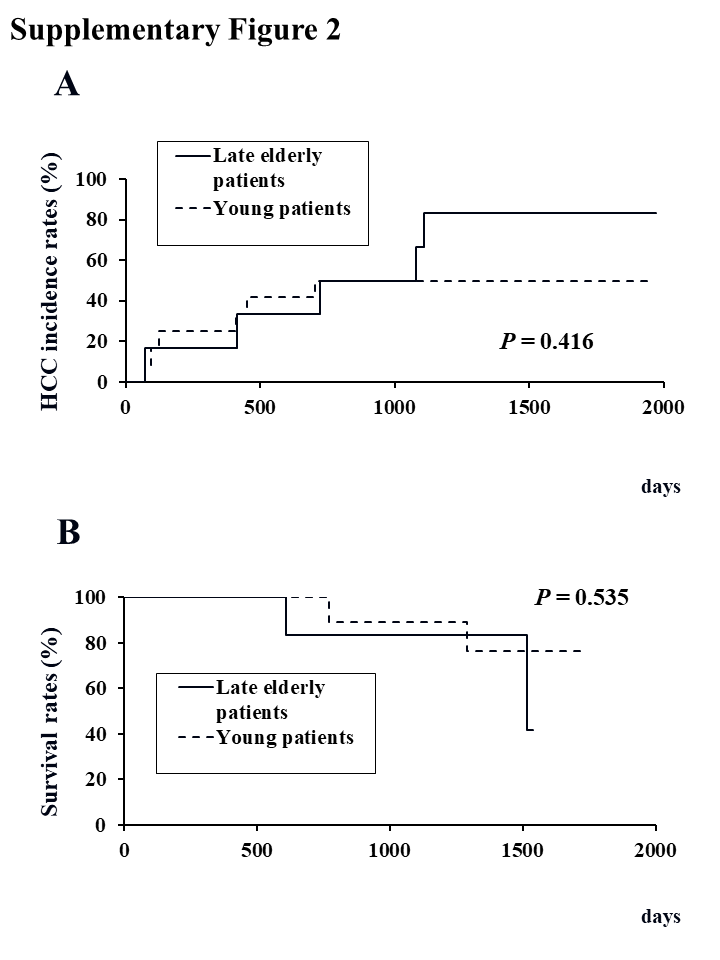

Supplement: Supplementary file 2 — Figure S2 (A) Cumulative hepatocellular carcinoma (HCC) incidence rates of the patients without HCC history. (B) Survival rates of the patients without HCC history after the start of direct‐acting antivirals (DAAs). No significant differences between late elderly patients and young patients were demonstrated in either the cumulative HCC incidence rates or cumulative survival rates. [file JGH3-5-122-s002.TIF]
